# Caregiver Perceptions of Communication About Early Cerebral Palsy or High-Risk Designation in Infants

**DOI:** 10.1001/jamanetworkopen.2025.19421

**Published:** 2025-07-08

**Authors:** Faith Kim, Sylvan Ryder, Arden Marin, Annette Zygmunt, Katherine Guttmann

**Affiliations:** 1 Department of Pediatrics, NewYork-Presbyterian Morgan Stanley Children’s Hospital/Columbia University Medical Center, New York; 2 Department of Pediatrics, Mount Sinai Kravis Children’s Hospital, New York, New York

## Abstract

**Importance:**

As early cerebral palsy (CP) detection becomes widespread, understanding caregiver perceptions about (1) early diagnosis or (2) high risk for CP (HRCP) designation is needed to identify areas for improvement in communication.

**Objective:**

To examine caregiver perceptions regarding clinician communication of a CP diagnosis or an HRCP designation in their high-risk infants.

**Design, Setting, and Participants:**

This prospective qualitative study was conducted from January 8 to October 25, 2024, at a level IV neonatal intensive care unit and high-risk infant follow-up program in an urban academic center. Caregivers were eligible for inclusion if (1) their infant received a CP diagnosis between January 3, 2022, and August 31, 2023, or (2) their infant received an HRCP designation but had at least 2 neuromotor assessments with normal findings by adjusted age 2 years or older with no CP diagnosis.

**Exposure:**

Caregivers’ exposure to conversations with clinicians regarding CP in their infant.

**Main Outcomes and Measures:**

Caregivers’ perceptions regarding a CP diagnosis and an HRCP designation were collected through in-person interviews, which underwent independent, directed, qualitative content analysis by 2 reviewers.

**Results:**

A total of 23 caregivers were interviewed (median [IQR] age, 34 [32-42] years; 19 women [82.6%]). The median (IQR) adjusted age of 12 infants at CP diagnosis was 6.07 (5.48-7.83) months and among 8 infants with an HRCP designation but no CP diagnosis, 7.50 (6.47-10.04) months. Four themes were identified: (1) degree of preparation for a future diagnosis or designation, (2) perceived negative communication practices, (3) caregivers’ emotional response to a diagnosis or designation, and (4) caregivers’ feedback on communication practices. Most caregivers recalled no previous CP counseling prior to a diagnosis or designation and often had a negative emotional response to these conversations. Caregivers in the CP group valued earlier diagnoses, while all but 1 in the HRCP group supported use of the designation but wanted subsequent conversations regarding when and whether the designation could be refuted.

**Conclusions and Relevance:**

In this qualitative study, most caregivers accepted earlier conversations regarding CP but preferred the use of positive language and provision of practical next steps with follow-up. These findings suggest that clinicians should prioritize early, directed conversations about CP when clinical suspicion arises and focus on infant’s strengths and time points for reevaluation.

## Key Points

**Question:**

What are caregivers’ perceptions regarding communication practices used to disclose a cerebral palsy (CP) diagnosis or high risk for CP designation in their infant?

**Findings:**

In this qualitative study involving in-depth interviews of 23 caregivers of high-risk infants, the majority accepted earlier conversations regarding CP, felt unprepared for conversations surrounding CP risk, recalled negative emotional reactions to a diagnosis or designation, and expressed wanting more practical next steps and follow-up.

**Meaning:**

These findings suggest that clinicians who suspect CP should prioritize early directed conversations; use clear, compassionate language; and provide explicit follow-up regarding the infant’s continued risk.

## Teaser

This qualitative study examines caregivers’ perceptions regarding clinician communication practices for disclosing a cerebral palsy diagnosis or a high risk for cerebral palsy designation for their infant.

## Introduction

Cerebral palsy (CP) is the most common motor disability in childhood, with 10 000 infants diagnosed annually in the US.^1,2,3,4,5^ Historically, clinicians perceived CP to be an unseen disability with a latent period before diagnosis.^6^ However, recent research has shown that validated tools and neuroimaging may predict CP in select infants as early as age 3 months, allowing for earlier, targeted interventions.^7^

Delayed diagnosis has been associated with caregiver dissatisfaction and depression, and caregivers have consistently identified early diagnosis as a priority.^8,9,10,11,12,13,14^ In a large early CP detection study, parents felt that clinicians demonstrated empathy while disclosing a diagnosis, but information was insufficient and terminology difficult to comprehend.^15^ Additionally, use of an interim high risk for CP (HRCP) designation is recommended for high-risk infants without a confirmed diagnosis to start early, goal-setting conversations and enable follow-up.^16^ Practice variability exists for difficult news disclosure, with little known about how to define and disclose HRCP. Improving understanding of caregivers’ perspectives may improve communication practices. The aim of this study was to explore caregiver perceptions of discussions with clinicians regarding a CP diagnosis or HRCP designation.

## Methods

This prospective, single-center, qualitative study was performed at an academic medical center with a level IV neonatal intensive care unit (NICU) and high-risk infant follow-up (HRIF) clinic in New York. This study was approved by the Columbia University Medical Center’s Institutional Review Board. The last treating clinician inquired about study participation, and if the caregiver expressed interest, a member of the research team obtained oral informed consent. The Standards for Reporting Qualitative Research (SRQR) checklist was followed.^17^

After we joined the Early Detection and Intervention Network supported by the Cerebral Palsy Foundation in December 2021, CP prediction tools were implemented in the NICU and clinic, including the General Movement Assessment (GMA) and Hammersmith Infant Neurological Examination (HINE). A CP diagnosis was considered if all the following were present: clinical risk factor, absent fidgety GMA, low HINE scores, neuroimaging or genetics correlated with CP, and gross motor impairment or considered later if longitudinal assessments repeatedly demonstrated motor impairment.^7^

Because a subset of infants followed up in the HRIF clinic did not have a previous GMA or HINE before early detection guidelines were implemented, we adopted a broader set of criteria, which were later modified once all tools were in use. During the study period, an infant was considered HRCP based on preestablished criteria (eTable in Supplement 1).^7,16^

The study was performed from January 8 to October 25, 2024. English-speaking caregivers were eligible if (1) their infant received a CP diagnosis between January 3, 2022, and August 31, 2023, or (2) their infant received an HRCP designation and had at least 2 reassuring neuromotor assessments with no CP diagnosis by adjusted age 2 years. Caregivers were contacted at least 3 months after a disclosure visit and needed to have access to a videoconferencing application. Interviews were open to 1 or 2 caregivers per family, and each participating family received a gift card at the end of the study. Enrollment continued until thematic saturation was reached, estimated at 10 caregivers each in the CP and HRCP groups among a relatively homogenous study population.^18^

Semistructured interviews were conducted by a neonatologist (F.K.), nurse practitioner (S.R.), psychologist (A.Z.), or pediatric resident (A.M.) using an interview guide created by the principal investigator (F.K.). F.K., S.R., and A.Z. practice in the HRIF clinic, while F.K. and A.M. practice in the NICU. To maintain reflexivity, caregivers were interviewed by a clinician most unfamiliar with their infant’s course and not directly involved in the disclosure visits.^19^ Interview prompts focused on caregiver experiences and responses to counseling about CP during the antenatal, NICU, and post-NICU periods. A mock interview was conducted with a volunteer parent of a preterm infant, and prompts were adjusted after each clinician interviewed at least 1 family. Audio recordings were converted into deidentified written transcripts using a Health Insurance Portability and Accountability Act–compliant Americans With Disabilities Act transcription service. Families were coded as either C or H to distinguish between the CP and HRCP groups. Demographic and clinical characteristics of infants were collected via electronic health record review. Median (IQR) adjusted age at CP diagnosis or HRCP designation with no diagnosis was reported in months. Self-reported race (participants identified as African American or Black [hereafter referred to as Black] or White) and ethnicity (participants identified as either Hispanic/Latinx or non-Hispanic) were extracted from the electronic health record or reported during the interview to delineate the representativeness of the population studied.

### Data Analysis

Two authors (F.K. and K.G.) conducted an independent, directed, qualitative content analysis of all deidentified interviews. Because F.K. played a primary role in implementing CP prediction tools, K.G., who is a neonatologist in a separate institution that does not use these tools, was the second coder to mitigate bias. As delineated by Braun and Clarke,^20^ we used a systematic process of coding to identify recurring patterns, which were then categorized into themes and subthemes.^21^ NVivo, version 14.23.2 (QSR International) qualitative analysis software was used to organize and code the data line by line and extract coded passages. A codebook was developed and continuously refined with iterative review until thematic saturation was reached, with consensus between both coders achieved.^22^

## Results

In the CP group, 15 caregivers from 12 families were interviewed, and in the HRCP group, 8 caregivers from 8 families were interviewed for a total of 23 caregivers (median [IQR] age, 34 [32-42] years; 4 men [17.4%] and 19 women [82.6%]; 13 identifying as Black [56.5%] and 10 as White [43.5%] race; 13 identifying as Hispanic/Latinx [56.5%] and 10 as non-Hispanic [43.5%] ethnicity) (Table 1). The highest level of education was at least college among 12 mothers (66.7%). Infant median adjusted age at CP diagnosis was 6.07 months (IQR, 5.48-7.83 months). Of the 12 infants with CP (6 boys and 6 girls), 6 (50.0%) were preterm, 5 (41.7%) were given an HRCP designation that converted to a diagnosis, 5 (41.7%) were diagnosed during or before their first HRIF visit, and 2 (16.7%) were diagnosed during a subsequent visit. Infant median adjusted age at HRCP designation in the HRCP group with no CP diagnosis (4 boys and 4 girls) was 7.50 months (IQR, 6.47-10.04 months). The following 4 themes were identified from the interviews: (1) degree of preparation for a future diagnosis or designation, (2) perceived negative communication practices, (3) caregivers’ emotional response to a diagnosis or designation, and (4) caregivers’ feedback on communication practices.

**Table 1.** Characteristics of Infants With CP or an HRCP Designation and Interviewed Caregivers

Interview No.	Infant characteristics									Caregiver characteristics	
	Gestational age, wk	Birth weight, g	Sex	Race	Ethnicity	CP risk factors	Age at diagnosis or HRCP designation^a^	Year of diagnosis or HRCP designation	CP type or other diagnosis (age)	Caregiver interviewed	Highest level of maternal education
**CP group**											
C1	26 2/7	1035	Male	White	Hispanic/Latinx	Prematurity, IVH	7 mo 23 d	2022	Spastic diplegia	Mother	College
C3	28 2/7	620	Male	White	Hispanic/Latinx	Prematurity, IVH	5 mo 20 d	2022	Spastic hemiplegia	Mother, father	Graduate
C4	35 2/7	1965	Female	Black	Non-Hispanic	Hemorrhagic infarctions	9 mo 11 d	2023	Spastic quadriplegia	Father	Less than high school
C5	26 6/7	624	Male	White	Hispanic/Latinx	Prematurity, FGR	24 mo 13 d	2023	Hypotonia	Mother	Graduate degree
C6	40 1/7	3815	Female	Black	Hispanic/Latinx	Dandy-Walker malformation	8 mo 29 d	2023	Hypotonia	Mother	College
C7	39 6/7	3660	Female	White	Non-Hispanic	HIE	12 mo 2 d	2023	Spastic hemiplegia	Father	Graduate degree
C10	40 4/7	3771	Female	Black	Non-Hispanic	Neuronal migration disorder	7 mo 7 d	2023	Spastic hemiplegia	Mother	High school
C11	40	4290	Male	Black	Non-Hispanic	HIE	3 mo 18 d	2023	Spastic diplegia	Mother, maternal grandmother	High school
C12	39 4/7	3235	Male	Black	Non-Hispanic	HIE	6 mo 22 d	2023	Spastic diplegia	Mother	High school
C13	23 4/7	600	Female	Black	Hispanic/Latinx	Prematurity	7 mo 7 d	2022	Spastic quadriplegia	Mother	College
C15	25	650	Male	Black	Hispanic/Latinx	Prematurity, FGR, PVL	12 mo 6 d	2023	Spastic diplegia	Mother	High school
C17	25 5/7	760	Female	White	Non-Hispanic	Prematurity, PVL	4 mo 3 d	2022	Spastic quadriplegia	Mother, father	College
**HRCP group**											
H3	30 3/7	1030	Female	White	Hispanic/Latinx	Prematurity	10 mo 14 d	2022	ASD (3 y 6 mo)	Mother	College
H5	24 5/7	530	Male	Black	Non-Hispanic	Prematurity	5 mo 16 d	2022	Speech delay (2 y 8 mo	Mother	College
H6	23 6/7	500	Female	Black	Hispanic/Latinx	Prematurity	6 mo 9 d	2022	Feeding issues (2 y 6 mo)	Mother	High school
H7	26 5/7	1105	Male	White	Hispanic/Latinx	Prematurity	5 mo 25 d	2022	Age-expected skills (3 y 2 mo)	Mother	College
H8	31 3/7	1560	Female	Black	Hispanic/Latinx	Prematurity, IVH	5 mo 9 d	2023	Speech delay (2 y 1 mo)	Mother	High school
H9	36 1/7	2180	Male	Black	Hispanic/Latinx	Prematurity	9 mo 16 d	2023	ASD (2 y 6 mo)	Mother	High school
H10	31 5/7	1430	Male	White	Hispanic/Latinx	Prematurity	3 mo 3 d	2023	Speech delay (2 y 6 mo)	Mother	College
H11	25 6/7	480	Female	Black	Non-Hispanic	Prematurity	7 mo 8 d	2021	Fine motor delay (4 y)	Mother	College

Abbreviations: ASD, autism spectrum disorder; CP, cerebral palsy; FGR, fetal growth restriction; HIE, hypoxic-ischemic encephalopathy; HRCP, high risk for cerebral palsy; IVH, intraventricular hemorrhage; PVL, periventricular leukomalacia.

^a^ Adjusted age if born preterm.

### Degree of Preparation for a Future Diagnosis or Designation

Subthemes included feeling informed or uninformed, suspicion that something was not right, and perceived role of HRIF (Table 2). Most caregivers could not recall previous CP counseling in the NICU and felt unprepared when these conversations eventually occurred. One caregiver felt frustrated because she wanted to know the range of outcomes: “I kept saying to [the NICU team], there’s something that you’re not telling us, just tell us so we know what we’re getting into, and we can prepare” (C11). Another caregiver wanted to be better prepared for these conversations: “I feel like they should have started preparing me for all this before we left the hospital vs just throwing it all at me at once in [HRIF]” (H10). One caregiver admitted that even if CP was mentioned in the NICU, she likely “tuned it out” (H5). Another caregiver of an infant with a brain malformation who had antenatal consults with neonatology, neurology, and neurosurgery felt informed about the range of developmental delays but expressed, “CP was something that I never heard of until HRIF; it was not something that we thought we would ever have to deal with” (C6). Half of the caregivers of infants with CP suspected that their infant’s motor development was delayed before a clinician disclosed a diagnosis. Few questioned the role of HRIF besides telling them that their child was behind developmentally instead of preparing them for next steps.

**Table 2.** Degree of Preparation for a Future Diagnosis or Designation

Subtheme	CP group (interview No.)	HRCP group (interview No.)
Feeling informed or uninformed	“I kept saying to them, there’s something that you’re not telling us, just tell us so we know what we’re getting into, and we can prepare. It wasn’t like, oh, well, he may never walk. Until this day, no one still has ever said that, because that’s something you really don’t know.” (C11) “Overall, everyone did a good job informing us. At the time we were like, ‘Why are they saying all the bad things that happen?’ But we realize they have to make us aware of all possibilities; it doesn’t mean that that’s what’s going to happen. I do feel like we were prepared, it didn’t change the fact that it was still tough and hard to go through. But CP was something that I never heard of until HRIF; it was not something that we thought we would ever have to deal with.” (C6)	“So cerebral palsy was never mentioned while he was in the NICU. Maybe it was, and I tuned it out. I’m not going to lie to you. I probably tuned it out like, ‘Oh, whatever, I don’t know what you’re talking about.’” (H5) Interviewer: “When would have been a good time to bring up [risk of CP in your child] with you?” Caregiver: “In the NICU…I feel like they should have started preparing me for all this before we left the hospital vs just throwing it all at me at once in [HRIF].” (H10)
Suspicion that something was not right	“I knew something was wrong because I’m not used to seeing children like that, but I don’t know what it was. When we knew that something was definitely wrong, she was 4 months, 5 months when she cannot sit. We do everything to sit her. If you sit her, she just dropped like a rock. I thought, maybe she’s too little for me to worry because she was born 2 months early. Maybe miracles are gonna happen. Maybe she is going to sit at 10 months or 1 year. But I knew something was wrong.” (C4) “And in my mind, I’m like, ‘He’s going to have some type of delay,’ but we don’t know. It’s no way. If he doesn’t, it’ll be a miracle. You can’t just be okay when you’ve had that type of trauma.” (C11)	“I was concerned because I have 3 other children, and I did see that he wouldn’t move, all my other children would move. I know they say not to compare, but it was inevitable for me. He wouldn’t smile, he wouldn’t make any facial expressions. He would barely move, so it was a bit concerning.” (H10) “I did notice [leg stiffness], but at the same time, I’m just thinking, okay, he’s going to develop late, so probably it’s not a big deal, especially since he was getting [physical therapy] at home.” (H5)
Perceived role of HRIF	“It feels more so being monitored and someone having eyes, where I feel that I don’t need eyes on top of my child in that way. It feels like an extra stressor for me to be constantly asked, what is he doing, what is he not doing, is he doing this, is he doing that? He is where he’s supposed to be, and he’s working at his own pace.” (C1) “I asked the team: ‘Can you guys just tell me what’s the point of me coming here? Because I know [she] is behind…we know she has CP, so why are we coming in to do evaluations for you guys to kind of reiterate the things that I already know?’ And they were kind of like, ‘No, we appreciate you asking that question. We want to monitor to know the progress she’s making, and then we are also here to help give you information if there’s any ways that we can help.’” (C13)	“After the second [HRIF] visit, they did the same exams and they kept saying he was delayed. But then his pediatrician would say, ‘No, he is on task and he’s doing what he needs to do.’ Because his problem is he doesn’t work on commands.…So then I feel like it wasn’t correctly scored because he wasn’t really giving his all. And I really didn’t understand any of it.” (H10) “I feel like [the HRIF clinic] helped me a lot through this journey because you guys catch stuff, me, as a parent, I cannot see.” (H8)

Abbreviations: CP, cerebral palsy; HRCP, high risk for cerebral palsy; HRIF, high-risk infant follow-up; NICU, neonatal intensive care unit.

### Perceived Negative Communication Practices

Subthemes included poor communication, focus on deficits, need for compassion, and navigating conflicting opinions (Table 3). Caregivers expressed the importance of considering the environment, including the number of people in the room, gauging caregivers’ understanding of their infant’s condition before delivering news, and offering to involve another parent or caregiver. One caregiver described a video visit with a non–HRIF clinician who diagnosed her son with CP within 15 minutes by saying, “He will never be able to play basketball, but he can be in a regular classroom” (C1). Another caregiver whose infant was in the NICU for months kept hearing the term CP during the first HRIF visit only to find out that her daughter’s previous records had cerebral palsy listed as a condition and kept asking the HRIF team, “Why do you keep saying CP? Are you telling me she has cerebral palsy?...No one had ever said CP to me” (C13). Caregivers often felt that clinicians focused on their infant’s deficits and worried that the assessments did not demonstrate their infant’s potential: “I don’t feel like she was being herself to get the right evaluation…when she’s home, I see one thing, and when we go to this 1-hour [HRIF] appointment, she’s not being herself; she’s just crying” (H8). Instead, caregivers wanted compassion from clinicians. Caregivers who reported a positive experience felt comfortable asking questions. Finally, several caregivers felt confused and even skeptical of the diagnosis or designation, especially when others did not agree: “His pediatrician would say no, he is on task and he’s doing what he needs to do” (H10).

**Table 3.** Perceived Negative Communication Practices

Subthemes	CP group (interview No.)	HRCP group (interview No.)
Poor communication	“She said, ‘He will never be able to play basketball, but he can be in a regular classroom,’ and it was like, wow. I’m a provider myself, and I would never speak in absolutes, even if I have an idea. It was a video call. We were not even in person. You didn’t even examine him. It was very matter of fact, nonchalant.” (C1) “They said, ‘Well, with the CP, with the CP,’ and then I said, ‘Why do you keep saying CP? Are you telling me that she has cerebral palsy?’ Then they realized that I had never been told that she has CP, and they were saying, we looked in her medical records. I don’t know if it directly says CP or if it alluded to it, but no one had ever said CP to me.” (C13)	“Doctors’ terminology uses very big words, sometimes they tell it to us and we’re just like, ‘What?’ We shut down. And I feel like everything was just like a bomb thrown at a person and then you have to receive it. And I was also going through postpartum depression, so it was all too much.” (H10) “But once [the doctors] realized that he didn’t have any brain bleeding, probably they didn’t need to touch base on that, if that makes sense. The main focus did become on his kidneys, ROP, and then the whole breathing and feeding himself. So I think probably that’s why they didn’t really touch on [risk of CP] as much, I’m assuming.” (H5)
Focus on deficits	“I’m mad at the moment because [the specialist] asked, ‘Oh, he can move, but he can’t do that but 3 months later, he can do everything.’ He can turn himself over.” (C3) “He was like, ‘She’s probably not going to walk.’ A doctor should never say something like that. You only can see one part. You don’t know everything that’s going on. For a doctor to tell you that definitively that she’ll never walk, who is he to say that? He’s not God.” (C17)	“I feel like he didn’t give his all and didn’t really show his full potential of what he does, and they scored him accordingly to whatever he showed.” (H10) “So, I don’t feel like she was being herself to get the right evaluation…when she’s home, I see one thing, and when we go to this 1-hour [HRIF] appointment, she’s not being herself, she’s just crying. She’s not comfortable.” (H8)
Need for compassion	“Some people are not considerate when telling parents something really heavy. Compassion has to come through because it’s heavy news, and we don’t always understand what we’re being told.” (C17) “The [HRIF team] was open and honest, but that is kind of what I was looking for in the NICU, but we didn’t get it till later.” (C11)	“I feel like sometimes doctors don’t have compassion towards the parent. They can’t feel what parents feel. But the delivery of things could have been different because I had no idea what [CP] was.” (H10) “I love the hospital and [HRIF] because I feel like I’m talking to family; I can say anything, I can ask questions with no judgment. Last time I was afraid to ask questions, but they told me, ‘Tell me what it is,’ and I feel comfortable.” (H3)
Navigating conflicting opinions	“It was a lot to take in. It was not something that I was expecting. [CP] was never mentioned to me and even now, I’m a little skeptical about that diagnosis for other reasons.” (C1) “Another way that it was explained was that having [CP] is more known and could help us get more resources. My pediatrician said it’s not something that’s going to hurt her by being on her record. So even if everyone is not in 100% agreement, it’s not going to change anything about her care at this point. I understood that; it made sense.” (C6)	“After the second visit, they did the same exams and kept saying he was delayed. But then his pediatrician would say, ‘No, he is on task and he’s doing what he needs to do.’ So I feel like it wasn’t correctly scored because he wasn’t giving his all.” (H10) “Once the doctor said he’s fine. I was like, okay. Bye. I’m never coming back. I never thought about [CP]. I never thought about it as it being an option or a possibility. I’m hoping that stays the same.” (H5)

Abbreviations: CP, cerebral palsy; HRCP, high risk for cerebral palsy; HRIF, high-risk infant follow-up; NICU, neonatal intensive care unit; ROP, retinopathy of prematurity.

### Caregivers’ Emotional Response to a Diagnosis or Designation 

Subthemes included negative perception, positive perception, fears for the future, and feeling unprepared for next steps (Table 4). Most caregivers recalled having a negative emotional response to conversations in which clinicians disclosed a diagnosis or designation, such as confusion, defeat, and shock, and often jumping to worst-case scenarios: “For me, everything was the same level of severity” (C15). One caregiver felt guilty after hearing that her infant was HRCP stating, “I felt like I was the one to blame…it was my fault that he was born early and that he could have CP” (H10). Several caregivers felt uninformed about next steps, expressing worries about their infant’s ability to walk even if the diagnosis was not definitive. Almost half of caregivers turned to other resources, such as online searches. They experienced information overload, which often left them feeling overwhelmed. Several caregivers admitted to not reading the resources provided to them at the HRIF, which included a book entitled *Cerebral Palsy Tool Kit: From Diagnosis to Understanding*^23^ and Cerebral Palsy Foundation websites.^24,25^

**Table 4.** Caregivers’ Emotional Response to a Diagnosis or Designation

Subthemes	CP group (interview No.)	HRCP group (interview No.)
Negative perception	“I was shocked because I was upset. Why didn’t I know about [CP], why didn’t anyone mention it to me? Am I behind on treatments that I should have been giving her? I left confused and upset and defeated.” (C13) “They spoke to me about the possibilities of [CP] being severe or not, but for me, everything was the same level of severity. I started learning more as he started growing, and I started talking to the doctors. But even though they would give me positive or negative outcomes, for me, it was the same result because I didn’t know what was going to happen in general.” (C15)	“I felt defeated. I felt scared. That’s just how I was feeling.” (H11) “I did leave distraught, there was no word. That was hard to hear, especially knowing everything I went through, the pregnancy, being in the NICU every day. That was the hardest part, the worst part. I felt like I was the one to blame. I felt like it was my fault that he was born early and that he could have had any CP or developmental delay. It got to a point where I would sleep with him because I was so terrified that something could happen or he could roll over and then wouldn’t be able to roll back.” (H10)
Positive perception	“I’m just hoping to figure out what to do to better his day-to-day because he’s gotten better in the health aspect, but I know that this is going to continue to be a thing that could progress or not progress. And I’m hoping that I can find the help that he needs to just make every day feel like normal and thoughtful for him, and not be defined by his disease or disorder.” (C5) “And seeing how well she was doing and how surprised the doctors were I felt like, okay, fine. If she gets a diagnosis, she’s doing such a good job, we’ll keep on working on this and keep on working through it.” (C7)	“It was helpful because I feel like as a parent, I was pushing myself a little more to help her develop. Come on, let’s do this. Let’s do that. I don’t know if that helped, but in my mind, as a parent, I was trying harder. In the back of my head, I was like, ‘Is she going to walk,’ or I was looking at different options, how I was going to deal with it....You look for more information, you understand, listen to how it really is, or she might need a wheelchair, or she might, you know, need more stuff…I was getting prepared for it.” (H8) “I think it was very helpful because I wouldn’t have imagined just not having that conversation and she having [CP]. That would have been traumatizing and very heartbreaking for me. But then, since I had the conversation and I was expecting that, in case that happened, I was going to get the support and help that I need. So now that it didn’t happen, I just feel very grateful and happy that my child is doing well.” (H11)
Fears for the future	“I asked them, ‘Will she walk? Will she talk? Will she do the things on her own,’ and they couldn’t tell me. They didn’t know, which I understand. You’re a parent getting this big news, and you want answers, and you want to know what this means, but there’s no definitive answer when you have a diagnosis like that.” (C17) “I went [online] fast. And then [social media], and started looking up CP. When I saw some of the children that have it, I’m just like, is that what’s going to happen to my son? Is that what I’m going to expect? Does he need a wheelchair? Does he need braces to help him walk? I only know from what I see and who I follow on [social media]. And that’s kind of, in a way, sad to say because it should be a provider who gives information on this.” (C5)	“I came home to [search online for] it because I had no idea what CP was. So to my understanding, he was going to be a vegetable. I didn’t even know that they had different stages of CP until I looked it up. That’s when I was like, ‘Oh, okay, so there’s different types. It’s not just automatically, boom, he’s going to be a vegetable, he’s not going to be able to walk or do anything for the rest of his life.’ I would have preferred more information as to his diagnosis and the different levels.” (H10) “I felt defeated. There were so many days that I didn’t know what the future holds for [her]. What our lives are going to look like. What if she doesn’t know how to walk? What if she doesn’t know how to talk? That was my main concern.” (H11)
Feeling unprepared for next steps	“I feel like even if a brochure would have been helpful. Or like, ‘Here’s our website, you can go read more.’” (C6) “[HRIFs] were helpful and referred me to places or offered that if I needed other services, to let them know. They did explain that CP doesn’t mean that he’s not going to walk but in case that he didn’t, they would provide me with information for him to have the support that he needed like a wheelchair or a walker.” (C15)	“I wish I had more from what and to what time they have risk of [CP]. Because I walked out like, ‘Okay, she could be 5 years old and she could still develop [CP].’” (H8) “It was handled well because [HRIF] told me do the steps. I was able to do it, get things that he needs for us to help him. It was great. I might be different than most parents because most find things very devastating, but I’m like, it’s okay. As long as he’s healthy.” (H9)

Abbreviations: CP, cerebral palsy; HRCP, high risk for cerebral palsy; HRIF, high-risk infant follow-up; NICU, neonatal intensive care unit.

### Caregivers’ Feedback on Communication Practices

Subthemes included timing of the conversation, impact of language, need for follow-up, and being an advocate (Table 5). Most caregivers in the CP group wanted the diagnosis even earlier and were open to starting these conversations in the NICU. One caregiver admitted, “I can’t remember in detail because a lot was going on. There was no way I was going to retain all that information” (H11). Another caregiver in the HRCP group supported having earlier conversations even if no diagnosis was made, saying, “You have to rip off the Band-Aid. It’s actually beneficial to let the parents know that [CP] can be a possibility…there’s never gonna be a right time to have it but let’s get this early” (H7). In terms of language used, one caregiver did not appreciate absolute statements and wanted to be seen in person for a diagnosis while another caregiver recalled, “When the first thing that comes out of your mouth is saying, ‘Well, he can’t do this, he can’t do that,’ you’re already setting me to be defensive as a parent” (C13). One family felt frustrated by an early diagnosis because of a lack of prognostic certainty they perceived during the disclosure visit: “The [HRIF clinician] said his brain is still young, so let’s wait and see, so why do you tell me you’re not sure? Don’t give early information to the parent if you’re not sure because you have to think about the family, the situation” (C3). Several caregivers conveyed that a timely follow-up visit would have been helpful rather than treating the disclosure visit as a one-time event. Finally, caregivers felt that they could better advocate for their infant with a diagnosis. One family whose daughter with periventricular leukomalacia was given a diagnosis before 3 months expressed, “It’s not the worst thing to have a CP diagnosis because you need to get the right things in place. It helped to know early and have the right people tell us what to do” (C17). Another family had conversations about CP starting in the NICU and felt that a diagnosis “just doubled down our results and made sure she had the therapy she needed. Being told about HRCP laid the groundwork” (C7).

**Table 5.** Caregivers’ Feedback on Communication Practice

Subthemes	CP group (interview No.)	HRCP group (interview No.)
Timing of conversation	“The [HRIF clinician] said his brain is still young, so let’s wait and see, so why do you tell me you’re not sure? Don’t give early information to the parent if you’re not sure because you have to think about the family, the situation.” (C3) “If medical data shows that she has cerebral palsy since birth, they should be able to tell me, ‘Unfortunately, your daughter is going to have cerebral palsy at a young age.’ But nobody told me that. Tell me. Don’t tell me some children are late in sitting. No, tell me straight…it’s not my fault. It’s how she was born.” (C4)	“It doesn’t even matter when you bring it up. That’s just a topic where you have to rip off the Band-Aid. And it’s actually beneficial to let the parents know that [CP] can be a possibility. Even when they told me, they didn’t make it seem like it was factual. They just said, ‘Listen, this may be a possibility,’ or ‘Let’s just get him checked out.’ So it is a conversation that needs to be had, and there’s never gonna be a right time to have it, but let’s get this early because they caught it early.” (H7) “There were conversations regarding CP [in the NICU] but I can’t remember in detail because a lot was going on. There was no way I was going to retain all that information. The [HRIF] clinic told me about CP and that could be something she may face. It happened at the appropriate time.” (H11)
Impact of language	“She just flat out said, ‘So kids who present with this, that is a symptom of CP.’ She said it just like that, a video call, which I thought was unprofessional, very inappropriate, I just didn’t feel like that was the way that you do things. We were confused.” (C1) “When the first thing that comes out of your mouth is saying, ‘Well, he can’t do this, he can’t do that,’ you’re already setting me to be defensive as a parent. But if that’s your only focus, then I felt like you’re not even coming from a strength-based nothing. You’re not even looking at the positive things or the things that are working well.” (C13)	“When they tell you cerebral palsy, as someone who’s not in the medical field, you think the worst. And the fact that then they said it was unspecified, it was like, okay, so I don’t know if he’s gonna be a vegetable.” (H10) “Hearing those encouraging words at her next [HRIF] visit, and people telling me that she is doing better, that was helpful. And it was very encouraging for me to keep moving and keep going.” (H11)
Need for follow-up	“Once I was calm and could process, I wanted more information. It could have been like, ‘I’m going to call you in a few days,’ and then in that call ask, ‘Hey, can I send you some info,’ or, ‘Can I point you to a website to get more information?’” (C6) “When I was in the NICU when they were explaining the brain bleeds, CP, anything that was on the table for her, should have been mentioned. They should have said ‘Hey, Mom, in your journey, this is kind of what you’re looking at in addition to her being premature and needing therapy, services, she might also with the brain bleed have developmental delays.’” (C13)	“We just never spoke about [CP] again. In my mind, it was gone.” (H9) “Nobody ever told me, ‘Okay, no, he doesn’t have this or anything.’ I’m still running with the same diagnosis [of HRCP] because nobody’s ever sat down and told me, ‘No, he doesn’t have it.’ They did say he was showing more improvement, but other than that, nobody ever told me if he was still under the diagnosis or not. To make sure he has or doesn’t have. I feel like that’s the biggest thing for me.” (H10)
Being an advocate	“We started talking to a social worker to see what are the things we should be doing like preparing our application to [early intervention], seeking out a physical therapist to hit the ground running when we came home. We found helpful [having a diagnosis] just doubled down our results and made sure she had the therapy she needed. Being told about high-risk [for CP] lay the groundwork. You really have to put in the effort and have the resolve to really get what you can for your child.” (C7) “It’s not the worst thing to have a CP diagnosis because you need to get the right things in place. It helped to know early and have the right people tell us what to do.” (C17)	“It’s really up to the parents to just be his advocate…and also if the doctors recommend something, and it’s just a recommendation. If they recommend something, you probably should just look into it. It doesn’t hurt to just look into it and take heed to what they’re saying because it may be helpful or beneficial in the long run.” (H5) “So when I heard it that time, it really stuck with me, and I was like, ‘Give me his PT in person…because that will help him.’ And one of you on your team even asked me for my case coordinator’s numbers so they can call. I think that it was handled really well because you guys told me do the steps. I was able to do it and get the things that he needs to help him.” (H9)

Abbreviations: CP, cerebral palsy; HRCP, high risk for cerebral palsy; HRIF, high-risk infant follow-up; NICU, neonatal intensive care unit; PT, physical therapy.

### Acceptance of an HRCP Designation With No CP Diagnosis

Caregivers in the HRCP group described a range of responses to the conversations in which an HRCP designation was disclosed from feeling empowered to understand the practicality of the designation to feeling confused. Though no infants in the HRCP group received a diagnosis of CP (by design), all but one caregiver supported its use. One caregiver described the conversation as “sucky, but I get it. It’s the reality of having a premature baby that [CP] could be a possibility, so I understood. I’m glad it did happen so we can kind of move on to the next phase” (H5). One caregiver felt confused by the designation and instead wanted conversations to start in the NICU, finding it hard to hear about her child’s risk for CP for the first time in HRIF.

Half of the caregivers were unaware at the time of the interviews whether their infant remained HRCP and assumed that CP was no longer considered because it was not mentioned again in HRIF. These caregivers were often told by clinicians that their child was progressing well. One caregiver said that CP was “never mentioned again, so I’m assuming because it wasn’t mentioned that we’re past that stage” (H5). Another stated that once she saw her son reaching more milestones, “In my mind, [CP] was gone” (H9). One mother described wondering whether her daughter’s risk would ever amount to a diagnosis as torture because CP was not discussed again in any of her 3 visits. The other half recalled feeling relieved after conversations in which a clinician stated that they were not worried about CP.

## Discussion

In this qualitative study, we explored the outcomes associated with clinician communication practices with caregivers of high-risk infants regarding a CP diagnosis and an HRCP designation. Despite increasingly early CP detection, best practices for communicating a CP diagnosis and an HRCP designation are not well-established, and many clinicians lack basic communication skills.^26,27^ Our work echoes previous findings that communication about CP can have a lasting impact on caregivers.^9,13,14,16^

Previous studies conducted before early CP detection reported similar findings. One study found that most parents were dissatisfied with diagnosis disclosure due to insufficient information or delay, while early disclosure along with empathy, transparency, and hope translated into increased satisfaction.^12,13,14,28,29,30^ Despite early CP diagnoses in our cohort, half of the caregivers suspected a delay, with caregivers wanting even earlier conversations and better transparency. Similar to what our caregivers experienced, clinicians in 1 study often focused on an infant’s deficits, rather than strengths, during counseling.^31^ Mixed caregiver reactions to a diagnosis have been reported, ranging from grief to relief.^9,10,14^ Disclosure of diagnoses such as CP and perinatal stroke has been associated with high rates of maternal anxiety, depression, and guilt.^32,33,34^ Understanding and evaluating the impact these conversations have on caregivers’ well-being may allow clinicians to provide better support.^35^

Our study explored caregivers’ experiences from the antenatal period through HRIF. Previous studies have explored experiences in the NICU, with caregivers often recalling negative memories and challenges with information retention.^9,36^ Multiple factors influence interactions with clinicians, including the caregiver’s emotional state and infant medical complexity.^37^ In our cohort, though several caregivers wanted to know about the possibility of CP before going to HRIF, only 4 could recollect CP being mentioned prior to NICU discharge.

Despite recommendations to discuss an HRCP designation with families, the criteria for designating an infant as high risk are not well delineated, and identification depends on availability of predictive tools and comfort with managing diagnostic uncertainty.^31^ The impact of language should also be considered. Experts have advocated the use of neutral language, such as possibility or probability of CP rather than risk, to avoid ableism, which may negatively impact patients by automatically regarding differences as deficits.^38,39,40^

The purpose of an HRCP designation needs to be clearly defined for caregivers, focusing on early transparent conversations that acknowledge uncertainty. In previous work, caregivers of terminally ill children felt that physicians found it difficult to speak freely about uncertainty, leading to postponed conversations and delayed decision-making, while another study found that clinicians’ inability to manage uncertainty led to caregiver distress.^41,42^ Though clinicians may worry that families will find them untrustworthy if they demonstrate uncertainty, most caregivers prefer that clinicians are upfront in acknowledging when a diagnosis or outcome may be incorrect.^42,43^

Caregivers rely on clinicians to provide knowledge and hope.^44^ An HRCP designation may promote such empowerment by addressing concerns early and creating a shared, longitudinal plan for follow-up.^16,45^ Caregivers in our study expressed acceptance of an HRCP designation despite its inherent uncertainty as long as its purpose was discussed with them, echoing a prior study in which caregivers of children with CP acknowledged that clinicians can make diagnostic errors.^16^ However, caregivers were clear that reevaluation and explicit conversations were essential, which mirrors a study that reported mixed feelings from caregivers who either made assumptions that no news was good news or found lack of follow-up to be distressing.^36^

### Limitations

This study had several limitations. First, the study was limited to English-speaking families. Second, caregivers who consented to participate in this study may have differed from nonparticipants. Third, we focused on caregivers’ perspectives, which are subject to recall bias. Fourth, clinicians may remember conversations differently. Fifth, interviews were conducted by multiple interviewers, which may have created inconsistencies. While we ensured that caregivers were interviewed by clinicians who were most unfamiliar with their infant’s clinical course, caregivers may have responded differently to clinicians with no NICU or HRIF background. Finally, HRCP criteria have evolved in our clinic to adhere more strictly to the consensus statement.^16,46,47^ Infants eligible for this study were limited to those who met previous criteria to maintain consistency, but this highlights the need for further standardization of the designation.

## Conclusions

In this qualitative study, caregivers recalled a range of emotions during discussions about CP with clinicians and prioritized early diagnoses coupled with language focused on strengths, next steps, and explicit follow-up. Although caregivers accepted the use of an HRCP designation, they expressed confusion regarding their infant’s continued risk. Clinicians should consider earlier conversations surrounding CP that acknowledge its inherent uncertainty, particularly in infants with an HRCP designation. Conversations should begin before NICU discharge and continue during outpatient treatment. Development of targeted CP- and HRCP-directed communication skills training and communication tools are important next steps toward improving caregivers’ experiences.

## Notes

Supplement 1. **eTable.** High Risk for Cerebral Palsy Designation Criteria
eReferences.

Supplement 2.
Data Sharing Statement
