# Supplementary material for: Caregiver Perceptions of Communication About Early Cerebral Palsy or High-Risk Designation in Infants
Source: JAMA Netw Open. 2025 Jul 8;8(7):e2519421. doi: 10.1001/jamanetworkopen.2025.19421 (PMC12238903; doi:10.1001/jamanetworkopen.2025.19421)
Supplement: Supplement 1. — eTable. High Risk for Cerebral Palsy Designation Criteria eReferences. [file jamanetwopen-e2519421-s001.pdf]

Supplemental Online Content

Kim F, Ryder S, Marin A, Zygmunt A, Guttmann K. Caregiver perceptions of communication about early cerebral palsy or high-risk designation in infants. *JAMA Netw Open*. 2025;8(7):e2518525. doi:10.1001/jamanetworkopen.2025.18525

eTable. High Risk for Cerebral Palsy Designation Criteria
eReferences

This supplemental material has been provided by the authors to give readers additional information about their work.

© 2025 Kim F et al. *JAMA Network Open*.

eTable. High Risk for Cerebral Palsy Designation Criteria

During the study period, an infant was considered HRCP if at least three of the following were present and were recommended to follow-up in three months for re-evaluation:
1) Clinical risk factor associated with CP[1]
2) Cramped synchronized and/or absent fidgety movements on the General Movement Assessment[1]
3) Hammersmith Infant Neurological Examination score below age expected values[1,2]
4) Gross motor impairment on the Alberta Infant Motor Scale (conducted in infants who not independently ambulating) or Bayley-4 and/or by therapist impression[1]
5) Neuroimaging associated with CP (e.g. high-grade intraventricular hemorrhage, cystic or diffuse periventricular leukomalacia, hypoxic-ischemic injury, perinatal stroke, neuronal migration disorders)[1]
6) Genetic biomarker associated with CP[3,4]

eReferences

[1] Novak I, Morgan C, Adde L, et al. Early, accurate diagnosis and early intervention in cerebral palsy: Advances in diagnosis and treatment. *JAMA Pediatr.* 2017;171(9):897-907.

[2] Romeo DM, Ricci D, Brogna C, Mercuri E. Use of the Hammersmith Infant Neurological Examination in infants with cerebral palsy: A critical review of the literature. *Dev Med Child Neurol*. 2016;58(3):240–245.

[3] Maitre NL, Byrne R, Duncan A, et al. "High-risk for cerebral palsy" designation: A clinical consensus statement. *J Pediatr Rehabil Med.* 2022;15(1):165–174.

[4] Gonzalez-Mantilla PJ, Hu Y, Myers SM, et al. Diagnostic yield of exome sequencing in cerebral palsy and implications for genetic testing guidelines: A systematic review and meta-analysis. *JAMA Pediatr*. 2023;177(5):472-478.

© 2025 Kim F et al. *JAMA Network Open*.
