# Supplementary material for: Caregiver Perceptions of Communication About Early Cerebral Palsy or High-Risk Designation in Infants
Source: JAMA Netw Open. 2025 Jul 8;8(7):e2519421. doi: 10.1001/jamanetworkopen.2025.19421 (PMC12238903; doi:10.1001/jamanetworkopen.2025.19421)
Supplement: Supplement 2. — Data Sharing Statement [file jamanetwopen-e2519421-s002.pdf]

Data Sharing Statement

Kim. Caregiver Perceptions of Communication About Early Cerebral Palsy or High-Risk Designation in Infants. *JAMA Netw Open*. Published July 08, 2025.
doi:10.1001/jamanetworkopen.2025.19421

Data
Data available: Yes
Data types: Data (not involving human participants)
How to access data: Fk2362@cumc.columbia.edu
When available: With publication

Supporting Documents
Document types: None

Additional Information
Who can access the data: Anyone requesting the data
Types of analyses: For research purposes
Mechanisms of data availability: With a signed data access agreement
